# Targetability of the neurovascular unit in inflammatory diseases of the central nervous system

**DOI:** 10.1111/imr.13121

**Published:** 2022-07-31

**Authors:** Brandon C. Smith, Rachel A. Tinkey, Benjamin C. Shaw, Jessica L. Williams

**Affiliations:** ^1^ Department of Neurosciences Lerner Research Institute, Cleveland Clinic Cleveland Ohio USA; ^2^ Department of Biological, Geological, and Environmental Sciences Cleveland State University Cleveland Ohio USA; ^3^ School of Biomedical Sciences Kent State University Kent Ohio USA; ^4^ Brain Health Research Institute, Kent State University Kent Ohio USA

**Keywords:** blood–brain barrier, endothelial cell, glia, neuroinflammatory disease, pericyte, tight junction

## Abstract

The blood–brain barrier (BBB) is a selectively permeable barrier separating the periphery from the central nervous system (CNS). The BBB restricts the flow of most material into and out of the CNS, including many drugs that could be used as potent therapies. BBB permeability is modulated by several cells that are collectively called the neurovascular unit (NVU). The NVU consists of specialized CNS endothelial cells (ECs), pericytes, astrocytes, microglia, and neurons. CNS ECs maintain a complex “seal” via tight junctions, forming the BBB; breakdown of these tight junctions leads to BBB disruption. Pericytes control the vascular flow within capillaries and help maintain the basal lamina. Astrocytes control much of the flow of material that has moved beyond the CNS EC layer and can form a secondary barrier under inflammatory conditions. Microglia survey the border of the NVU for noxious material. Neuronal activity also plays a role in the maintenance of the BBB. Since astrocytes, pericytes, microglia, and neurons are all able to modulate the permeability of the BBB, understating the complex contributions of each member of the NVU will potentially uncover novel and effective methods for delivery of neurotherapies to the CNS.

## 1 INTRODUCTION

The blood–brain barrier (BBB) is a complex, highly regulated system with multiple cell types influencing its maintenance and formation. The BBB forms a privileged environment for the central nervous system (CNS), restricting entry of a wide breadth of potential hazards, including pathogens, immune cells, antibodies, and pharmaceuticals. This tight regulation protects the brain from external insults, but simultaneously prevents access of many therapeutics meant to treat neuroinflammatory and neurodegenerative disorders. As a result, multiple clinical trials have failed despite promise in preclinical studies, underscoring the need for a more complete understanding of the BBB and its modulatory mechanisms. In this review, we discuss the composition of the neurovascular unit (NVU), known mechanisms of BBB modulation, and potential therapeutic targets for neuroinflammatory disorders.

The existence of a selectively permeable BBB was first postulated at the turn of the 20th century when it was noted that water‐soluble dyes injected into the periphery did not permeate the CNS,^1^, ^2^ and dyes injected into the CNS parenchyma did not exit to the periphery.^2^, ^3^ Further complexity of the BBB was demonstrated by seminal evidence indicating immune privilege, wherein immune cells are excluded and unable to surveille the CNS, over 100 years ago by Shirai^4^ and later confirmed by Murphy and Sturm.^5^ However, even now, the permeability, complexity, and dynamics of the BBB are incompletely understood.^6^, ^7^

The BBB acts as a robust biological gateway, responsible for the flow of nearly all molecules and cells passing into and out of the CNS.^6^, ^8^, ^9^ This selective permeability is critical in sparing the CNS from many circulating toxins and pathogens and allowing for availability of necessary nutrients to the CNS.^2^, ^10^, ^11^ Instead of passing through fenestrations in endothelial cells (ECs) as it does in the peripheral vasculature, material is transported and scrutinized by one or more cells closely associated with the BBB.^11^, ^12^ However, it is this very system, designed to protect the CNS, that also hinders therapeutic development to treat CNS disorders including multiple sclerosis (MS), Alzheimer's disease (AD), Parkinson's disease, traumatic brain injuries, and brain cancers.^10^, ^11^, ^13^ Several researchers have even employed artificial intelligence to determine the likelihood of small molecules to penetrate the BBB.^13^, ^14^

This review will examine some of the NVU cell‐specific effects on BBB permeability as well as the intercellular communication that is critical for proper function of the NVU. While other complex CNS barriers, including the blood‐retinal barrier, the blood‐cerebrospinal fluid barrier, and the arachnoid layer,^15^ are also important to consider in therapeutic availability to the CNS, we will only focus on the BBB for the purposes of this review.

## 2 STRUCTURE AND ORGANIZATION OF THE NVU

**FIGURE 1:**
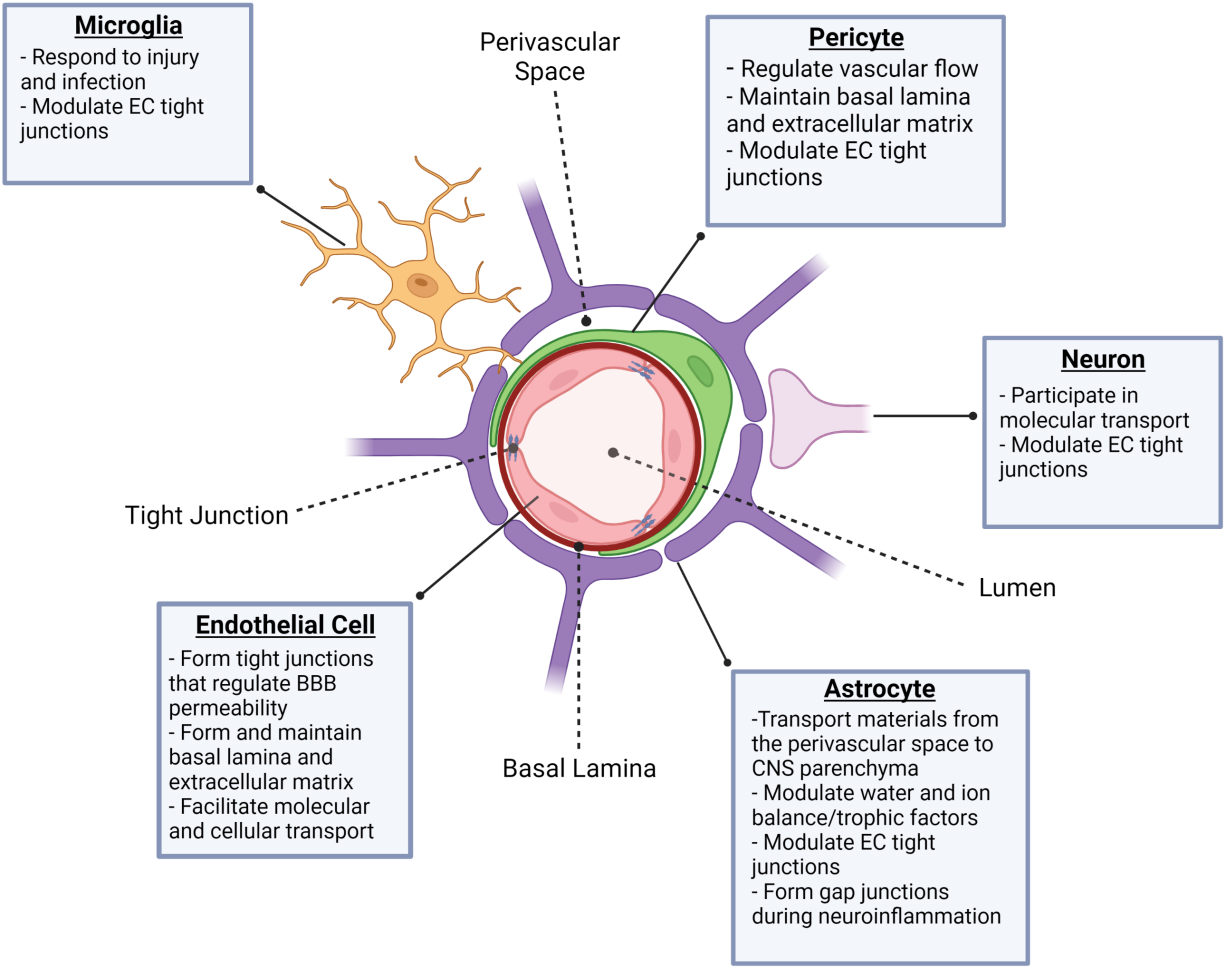
Cellular components of the neurovascular unit. The NVU is composed of a complex network of cells that are functionally diverse. ECs form the walls of blood vessels and capillaries and contribute to the formation and maintenance of the basal lamina and extracellular matrix. In addition, tight junctions formed between ECs and expression of adhesion molecules regulate BBB permeability. Pericytes reside in the capillary bed and, with regard to BBB integrity, are primarily responsible for modulation of vascular flow as well as structural changes in tight junctions and the extracellular matrix. Astrocyte endfeet cover 90%–95% of the area surrounding the BBB and contribute to a variety of processes that include, but are not limited to, osmotic homeostasis, trophic factor concentration, molecular transport into and out of the perivascular space, and formation of gap junctions under neuroinflammatory conditions. Microglia have been found to cover the remaining space around BBB ECs, respond to injury and infection, and regulate tight junction formation between ECs. Neurons predominantly communicate with astrocyte endfeet and aid in regulation of tight junctions and molecular transport.

The NVU is the fundamental multicellular unit supporting the BBB and consists of ECs, pericytes, astrocytes, microglia, and neurons (Figure 1).^6^, ^7^ The capillary lumen is the primary area of contact between the NVU and the periphery, and interfacing factors vary from cytokines and hormones to immune cells that have the potential to extravasate.^16^, ^17^ Forming the vessel lumen is specialized CNS ECs, which are semipermeable under homeostatic conditions due to the presence of tight junctions.^6^, ^16^, ^18^, ^19^, ^20^ Perivascular mural cells, or pericytes, have recently been shown to be more complex and varied than initially assumed. Pericytes are functionally heterogeneous^21^, ^22^ and further understanding of their diverse roles in the CNS is an active area of research. Pericytes were classically described to help govern vasoconstriction and dilation of capillaries,^23^, ^24^ which is highly regulated in the NVU, to limit BBB permeability. Recent studies are beginning to uncover pericyte coordination with other members of the NVU, particularly ECs and astrocytes.^21^, ^24^ Astrocytes are vital to the NVU and while the other members of the NVU have critical roles, it is largely the astrocytes that set the NVU apart from other capillary layers in the body.^25^, ^26^ Understanding the specific mechanisms by which astrocytes regulate tight junctions in CNS ECs could prove incredibly beneficial to optimizing BBB permeability for drug delivery. Astrocytes are relatively large and abundant CNS trophic cells^27^ and their endfeet form an additional barrier around CNS capillaries.^6^, ^25^ The perivascular, or Virchow‐Robin, space refers to the area between ECs and astrocytes and is a key checkpoint prior to entering the CNS parenchyma.^6^, ^28^ Microglia are a recent addition to our understanding of the NVU. Although astrocytes cover the majority of the perivascular space, there are some gaps in coverage. Recently, Kisler et al. used two‐photon in vivo imaging to observe microglial processes covering many of these gaps.^29^ How involved microglia are in the structure of the NVU is still being investigated. By area, neurons have a relatively minor role in the structure of the NVU but can modulate BBB permeability via neuronal activity.^30^, ^31^ Due to the complex nature of the NVU, it has proven difficult to study. In vitro models have been useful; however, the heterogeneity between species, individuals, and even CNS regions have added to this investigational barrier.^32^, ^33^ Here, we will discuss the components of the NVU and how each cell type may impact BBB permeability.

## 3 ENDOTHELIAL CELLS

ECs are found throughout the body and make up the walls of arteries, veins, and capillaries. They form a tube‐like structure to allow passage of various blood cells and proteins.^34^ ECs secrete and maintain the basal lamina—the extracellular matrix on which they reside.^35^, ^36^ The CNS ECs differ in several ways from peripheral ECs. Most notably, they lack the fenestrations found in peripheral vessels and instead create a continuous cellular barrier with significantly reduced permeability along the capillary lumen.^37^ Instead, CNS ECs form tight junctions which prevent hydrophobic molecules from penetrating the endothelial layer unless transported through the cell. These tight junctions are responsible for much of the impermeability of the BBB. Tight junctions are composed primarily of claudin‐5, occludin, and other junctional adhesion molecules^38^ and bind to the actin cytoskeleton via ZO‐1, ZO‐2, or ZO‐3.^39^ Tight junctions are highly complex and can vary depending on the proteins coupled.^40^, ^41^ Transport of nutrients, waste, and signaling molecules between the CNS and the periphery is necessary under physiological conditions and is typically achieved by a myriad of EC transporters.^6^, ^42^ In fact, these transporters make up 10–15% of the total protein in CNS ECs and allow specific molecules, peptides, and even cells to cross into the perivascular space, bypassing tight junctions.^33^, ^43^

### 3.1 ECs and immunity

Classically, it was thought that immune cells could not access the CNS parenchyma.^18^, ^44^ While mostly impermeable during homeostatic conditions, CD4^+^ and CD8^+^ T cells are allowed passage for immune surveillance of the CNS, facilitated by a highly regulated, multistep process through a tricellular junction.^45^ This process involves endothelial ligands vascular cell adhesion molecule (VCAM)‐1 and intercellular adhesion molecule (ICAM)‐1 which recognize lymphocyte function‐associated antigen 1 (LFA‐1) and very late antigen‐4 (VLA‐4) on T cells. These interactions arrest T cells on the endothelium and allow migration against the flow of blood to these tricellular junctions and ultimately extravasation.^45^, ^46^ These tricellular junctions contain the proteins tricellulin and angulin‐1 which direct and aid T‐cell diapedesis.^45^ While CNS ECs express VCAM‐1 and ICAM‐1 under normal conditions, the expression is sparse, limiting the number of leukocytes that can traverse tricellular junctions.^45^, ^47^

In response to infection, autoimmunity, or injury, the permeability of the BBB is significantly enhanced and can lead to severe inflammation.^48^ This is partially due to an increase in the expression of adhesion molecules on the EC surface, primarily ICAM‐1 and VCAM‐1, which can arrest a greater number of immune cells for extravasation.^49^ Inflammation can lead to break down of the tight junctions, allowing leukocytes to invade the perivascular space.^48^ Many secreted inflammatory factors, cell damage signals, and pathogen components can alter tight junction integrity including CCL2 and transforming growth factor (TGF)‐β, which is known to alter expression of claudin‐5, occludin, and ZO‐1.^39^, ^50^, ^51^ Likewise, tumor necrosis factor (TNF)‐α, lipopolysaccharide (LPS), and mitochondrial damage can induce BBB permeability via actin filament rearrangement.^52^, ^53^, ^54^, ^55^ Prolonged dysfunction of the BBB, as in chronic inflammatory CNS diseases such as MS or AD, can lead to permanent CNS tissue damage and neuroaxonal loss.^56^, ^57^, ^58^ However, although overt and/or chronic inflammation can lead to the loss of BBB integrity and extensive damage to the CNS, low‐to‐moderate amounts of inflammation can partially restore the BBB and limit peripheral immune cell infiltration into the CNS parenchyma, mitigating injury, or infection.^56^, ^59^, ^60^, ^61^ Taking advantage of this immunological state and exploiting molecular signals used in the formation of tricellular junctions may be a novel avenue for therapeutic development.

### 3.2 Targeting ECs

It is possible to permeate the BBB to treat neurological disorders, but the potential bystander effects of leaky BBB during diseases such as glioblastoma, MS, AD, and others imposes significant risk. The BBB is disrupted in many neurodegenerative and psychological disorders. Additional access of immune cells, toxins, or other inflammatory mediators could enhance inflammation‐mediated damage to the CNS. Additionally, BBB dysregulation disrupts homeostatic transport across the barrier, which provides trophic factors and controls osmotic regulation during physiological conditions.^62^ Bypassing the ECs of the BBB is a difficult challenge; however, there are several other members of the NVU that introduce potentially novel avenues for drug targeting.

## 4 PERICYTES

Proportionally, there is a higher density of pericytes in the CNS relative to peripheral organs,^24^ suggesting they have a critical role in the CNS. As vascular mural cells, pericytes play a large role in the dilation of capillaries in the NVU. Of note, pericytes are found in the capillary bed and not surrounding arteries or veins, which house similar, but distinct, vascular mural cells, and vascular smooth muscle cells.^63^ In addition to vasodilation and vasoconstriction, pericytes also help form and maintain the basal lamina.^64^, ^65^ Control of vascular flow by pericytes can indirectly impact the BBB as changes in blood flow can allow for more or less cellular contact in the capillary lumen. Increases in cellular interaction can stretch and stress CNS ECs,^66^, ^67^, ^68^ causing pericyte dysfunction, which is associated with aberrant immune cell trafficking.^69^ Further, in mouse models deficient in functional pericytes, there is a loss of vascular control, dysfunctional tight junction regulation, and aberrant angiogenic sprouting, highlighting the importance of pericytes in maintaining a healthy BBB.^69^, ^70^, ^71^, ^72^, ^73^

**FIGURE 2:**
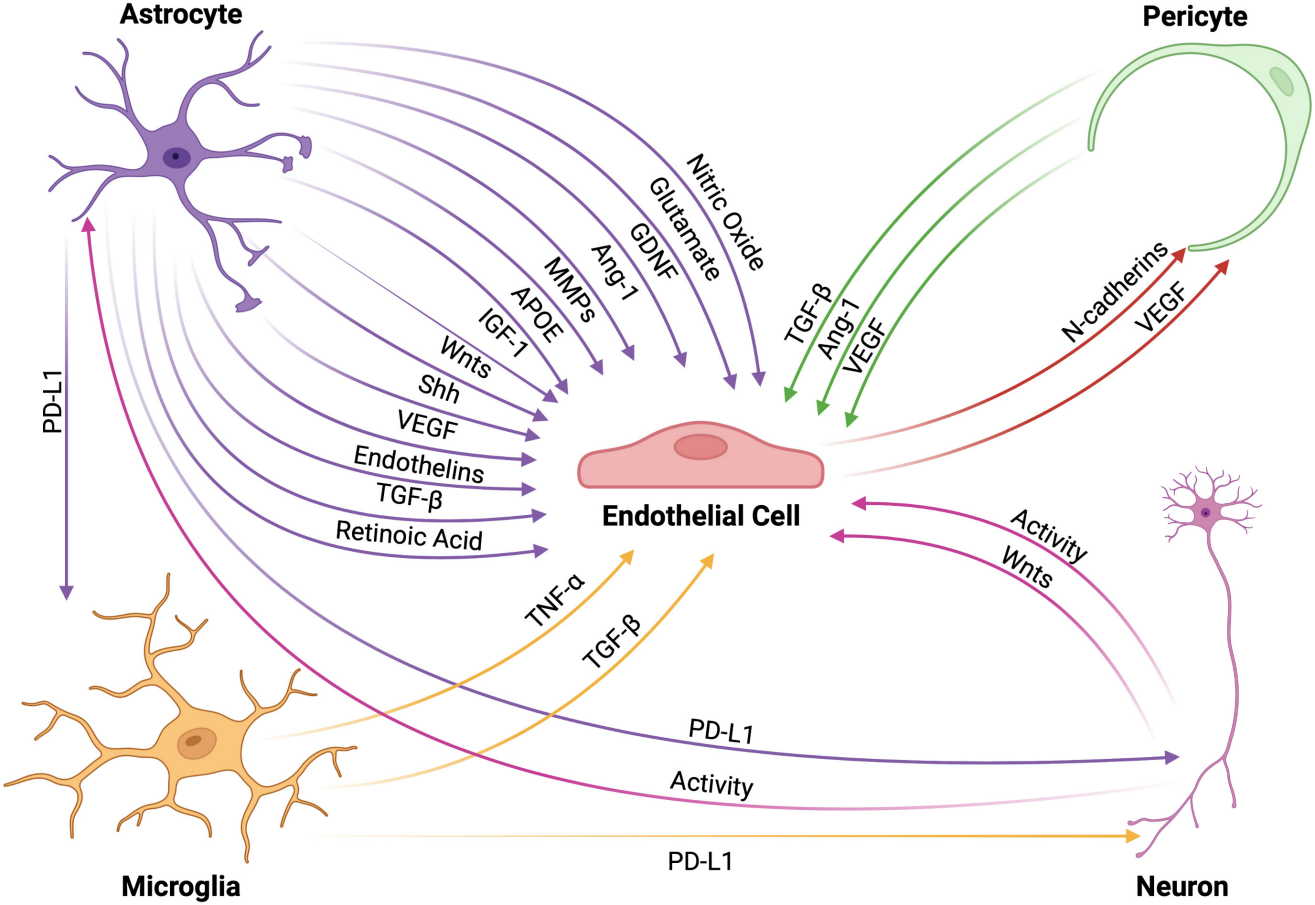
NVU interaction network. Cells within the NVU interact through intricate signaling mechanisms that allow for proper functioning of the BBB. ECs receive a variety of protective signals from other NVU cells that upregulate tight junction formation thus enhancing BBB integrity. These factors include, but are not limited to, TGFβ, Ang‐1, APOE, Shh, Wnts, glial‐derived neurotrophic factor, insulin‐like growth factor (IGF)‐1, and retinoic acid. Contrastingly, ECs may also receive signals that downregulate tight junction proteins, particularly during inflammatory events, such as TNFα, NO, MMPs, endothelins, and glutamate that lead to increased BBB permeability. Importantly, ECs also maintain the ability to signal to pericytes through VEGF and N‐cadherin, which are necessary for BBB maintenance. Immune checkpoint proteins, such as the PD‐1/PD‐L1 complex, regulate the cellular activity of microglia and neurons to modulate BBB integrity as well as dampen the inflammatory activity of infiltrating peripheral immune cells, which increases BBB permeability. Finally, neuronal activity is a critical modulator of cellular and molecular transport across the BBB.

New roles for pericytes continue to be uncovered, including their ability to maintain and produce elements of the basal lamina^74^, ^75^, ^76^, ^77^ and intricate regulation of tight junctions. Contrary to ECs, TGF‐β and angiopoietin‐1 (Ang‐1) signaling in pericytes enhances the expression of occludin on CNS ECs, reducing the permeability of the BBB. TGF‐β from pericytes also activates Smad4 signaling to upregulate bone morphogenic proteins (BMPs). BMPs can then ensure tight adherence of pericytes to ECs via N‐cadherins, which is upregulated by vascular endothelial growth factor (VEGF), reinforcing the communication and physical interaction between pericytes and CNS ECs.^78^, ^79^, ^80^ While basal levels of VEGF signaling can reduce BBB permeability, excessive VEGF signaling to pericytes can lead to downregulation of claudin‐5 on ECs and dysregulate BBB tight junctions (Figure 2).^81^, ^82^, ^83^, ^84^

### 4.1 Heterogeneity of pericytes

Similar to ECs, pericytes respond robustly to various signaling molecules. Inflammatory and non‐inflammatory pericytes have been described and subdivided into Type‐1 pericytes (PC1) and Type‐2 pericytes (PC2). PC1s are non‐inflammatory and tend to be the resting state of pericytes without injury or infection. PC2s tend to increase in frequency with and are highly responsive to inflammation.^73^, ^85^ It is likely, as is the case with astrocytes and microglia that there is a spectrum of activation states, but as the study of pericytes is still in its infancy, this has not yet been fully elucidated. In young healthy patients, nearly all pericytes exhibit a non‐inflammatory morphology.^71^, ^85^, ^86^ However, aging results in an increase in the population of inflammatory pericytes, which is consistent with enhanced inflammation and BBB permeability associated with age.^85^ Both pericyte subtypes contribute to the basal lamina, but PC2s tend to produce a more irregular basal lamina, impacting both ECs and astrocytes.^21^, ^85^ Additionally, PC2s produce less laminin‐111 and laminin‐211, which leads to cell hypertrophy and BBB disruption.^87^, ^88^ Exposing pericytes to inflammatory cytokines, LPS, or reactive oxygen species induces immunoreactivity in pericytes, altering their morphology, as well as inducing their separation from the basal lamina.^87^, ^88^, ^89^ The dynamic morphology of pericytes suggests they have a critical role in the integrity and function of the BBB, which is altered in pathological states and in aging.

### 4.2 Targeting pericytes

Examining the targetability of pericytes and its potential influence on the BBB is difficult but may prove promising. Affecting pericytes may be a subtler approach to drug penetrance rather than reducing the integrity of the EC layer itself. A deeper understanding of how pericytes communicate with CNS ECs and astrocytes could reveal potential nuanced approaches to bypass the BBB. While pericytes may be a promising target to leverage BBB permeability, much about pericyte mechanisms of BBB control remains unknown.

## 5 ASTROCYTES

Astrocytes are large stellate cells with extensive processes that extend throughout the CNS.^27^, ^90^ However, unlike CNS ECs and pericytes, astrocytes are unique in that only the ends of their processes are considered part of the NVU (Figure 1). These endfeet cover roughly 90–95% of the area surrounding the BBB and have properties that are unique from the rest of the astrocyte.^91^, ^92^, ^93^ Endfeet contain aquaporin‐4 and the potassium channel K_ir_4.1 to modulate water and ion balance and, lacking tight junctions, astrocytic endfeet permit immune cell extravasation.^92^, ^94^, ^95^ Although endfeet lack the barrier that tight junctions provide, ablation of astrocytes leads to rapid and extensive BBB deterioration.^25^, ^93^ And interestingly, early studies transplanting astrocytes outside of the CNS demonstrated that transplanted astrocytes develop a BBB‐like morphology surrounding the peripheral vasculature.^96^, ^97^ In addition to the physical barrier that astrocytes provide, during homeostasis, astrocytes are responsible for the transport of material from the perivascular space into the parenchyma.^6^, ^25^ This function makes them excellent and critical supportive cells in the maintenance of the BBB.

Astrocytes form gap junctions between endfeet forming a much “looser” network of connections compared with that of CNS ECs.^98^ Gap junctions are formed by a hexamer of proteins called connexins. The material that is transported by a given gap junction is largely dependent on the specific combination of connexins that make up the junction.^99^, ^100^ Notable gap junctions in astrocytes are formed by connexin 30 and connexin 43, which help mediate glucose and lactate transport to distal neurons.^101^ Astrocytes use these gap junctions to communicate with other cells of the NVU including neurons, microglia, and other astrocytes using ion gradients, electrical signals, and signaling molecules.^102^

### 5.1 Tight junction modulation

Astrocytes release a variety of trophic factors that help maintain a functional NVU.^103^, ^104^, ^105^ Many of the factors released under physiologic conditions increase the amount and order of tight junction proteins between BBB ECs.^106^, ^107^ For instance, astrocytes secrete sonic hedgehog (Shh), which increases tight junctions in CNS ECs by inducing Patch1 signaling.^107^ Other astrocytic factors that can enhance tight junctions in ECs include Wnt signaling, TGFβ, and apolipoprotein E (ApoE).^104^, ^106^, ^108^, ^109^, ^110^ ApoE and TGFβ may work indirectly through pericytes to impact EC tight junctions.^111^, ^112^ Additionally, astrocytes produce Ang‐1, which signals to EC Tie2 to further upregulate tight junction proteins and reduce adhesion molecules, reducing the likelihood of leukocyte entry.^113^, ^114^ Further, astrocytes are incredibly responsive to inflammatory stimuli,^115^, ^116^ and during inflammation, it has been shown that BBB ECs downregulate claudin‐5, while astrocytes upregulate tight junction proteins claudin‐1, claudin‐4, and JAM‐A.^117^, ^118^ This suggests that astrocyte endfeet form a secondary barrier to prevent excessive immune cell entry past the perivascular space, regulating access to the CNS parenchyma.^117^ This accumulation of peripheral immune cells in the perivascular space is often referred to as perivascular cuffing and is commonly seen in MS lesions.^117^, ^119^

However, although astrocytes provide an impressive physical barrier, activated astrocytes can also secrete trophic factors, chemokines, and cytokines that dysregulate EC tight junctions and recruit peripheral immune cells (Figure 2).^25^, ^39^, ^119^, ^120^ These factors can include VEGF, nitric oxide, MMPs, endothelins, and glutamate; all of which can downregulate endothelial tight junction proteins.^39^, ^121^, ^122^ Importantly, astrocytes also promote a return to homeostasis following an inflammatory event, secreting several beneficial trophic factors including Shh, astrocyte‐derived Ang‐1, glial‐derived neurotrophic factor (GDNF), insulin‐like growth factor‐1, ApoE, and retinoic acid. These factors not only prevent EC apoptosis, but also stimulate tight junction formation and a return to a homeostatic state.^116^, ^121^ Taken together, astrocytes orchestrate a complex modulation of the BBB and may represent an amenable cell type for drug manipulation.

### 5.2 Astrocytes as an immunologic barrier

Since astrocytes are highly responsive to immune stimuli, they have the ability to upregulate a wide array of chemokines and adhesion molecules that can serve as an immunologic barrier to prevent excessive influx of inflammatory cells during a CNS insult (Figure 2).^120^, ^123^ Interestingly, in the event immune cells breach the BBB, enter the perivascular space, and cross astrocyte endfeet into the parenchyma, astrocytes can express immune checkpoint molecules, or inhibitory receptors, which can induce exhaustion and death of leukocytes,^124^, ^125^ providing the CNS multiple layers of immunologic protection. As an example, astrocytes are known to upregulate the immune checkpoint protein programmed death ligand 1 (PD‐L1) in response to inflammatory cytokines, primarily interferons.^126^ PD‐L1 typically signals to cells via programmed death 1 (PD‐1) to reduce activation and induce apoptosis.^127^, ^128^ While many CNS cells, including microglia and even neurons, express PD‐1, it is highly enriched on immune cells, making them particularly susceptible to exhaustion.^126^, ^127^, ^128^, ^129^ In summary, astrocytes act not only as a physical barrier, but also a trophic and immunologic barrier to limit and control the activation state of immune cells that enter the CNS during neuroinflammation.

### 5.3 Targeting astrocytes

Astrocytes have great potential as a therapeutic target for BBB maintenance. Their ability to weaken EC tight junctions while maintaining a barrier could prove beneficial in the development of drugs to bypass traditional CNS barriers. There are many examples of astrocytes guiding various cell types and molecules across the BBB. This principle—if better understood—could be leveraged to advance CNS drug permeability. Similar to pericytes, astrocytes have great influence on the maintenance of EC tight junctions. Elucidating the molecular control that astrocytes have over BBB permeability will inevitably lead to more avenues of drug delivery.

## 6 MICROGLIA

Microglia are the resident immune cells of the CNS. As such, their functions are multifaceted. While they share many similarities with peripheral macrophages, they have many distinct characteristics, including their origins. Macrophages are generated in the bone marrow and circulate in the blood, while microglia migrate from the embryonic yolk sac.^130^ In general, microglia are responsible for initial responses to injury and infection, clearance of waste, synaptic pruning and maintenance, as well as providing several trophic factors to other cells of the NVU.^130^ Although it has been long appreciated that microglia is vital to the health of the CNS, surveying along the BBB and responding quickly to breaches, they are a relatively recent addition to the NVU and provide coverage of BBB areas not wrapped by astrocytes.^29^, ^131^ In addition, microglia are known to signal to other members of the NVU and communicate with peripheral immune cells,^6^ attracting and/or activating them within the CNS parenchyma during injury and infection as sentinels of the CNS.^132^, ^133^, ^134^, ^135^ Activated microglia and peripheral immune cells can secrete a number of factors that modulate BBB integrity (Figure 2). This is discussed in a number of reviews, but as an example, TNF‐α and TGF‐β can be secreted from reactive microglia under certain conditions which can either increase or decrease BBB integrity, respectively.^123^, ^136^, ^137^ While ablation or depletion of microglia did not result in overt BBB breakdown,^138^ recently, microglia was described to intricately associate with CNS capillaries and contribute to blood flow regulation and vasodilation.^139^, ^140^, ^141^ Additionally, microglia are incredibly motile and migrate quickly in response to injury, BBB leakage, and/or inflammation.^142^ Finally, similar to astrocytes, microglia can upregulate PD‐L1 in response to neuroinflammation,^143^, ^144^ providing multiple mechanisms of potential control of the BBB by microglia, although there is still much to learn about the intricacies of microglia and their impact on BBB permeability.

Given their emerging role in the NVU, the mechanisms underlying microglial regulation of the BBB remain incompletely described. Targeting microglia may modify CNS capillary permeability or other NVU cells to temporarily allow access to the CNS, but there is still much to learn about this exciting new potential target.

## 7 NEURONS

Neurons are the functional unit of the CNS and most other cells in the CNS support them, either directly or indirectly.^27^ Despite their indispensable role in the CNS, as a member of the NVU, they do not directly provide a physical barrier, but instead release a number of factors that modulate other NVU cells^27^, ^145^ using primarily their axons and dendrites.^6^ Interestingly, like astrocytes, neurons can induce a BBB‐like barrier in neighboring ECs,^96^, ^97^ suggesting neurons influence the formation and maintenance of the BBB in vivo.^146^, ^147^ Astrocytes and neurons are highly communicative,^148^, ^149^ providing a potential route of BBB control. Additionally, neural activity can influence the integrity of the BBB,^150^, ^151^, ^152^ although it is unclear if neural activity directly impacts ECs or if neuronal signals are propagated through astrocytes.^153^

Neuroinflammation can alter the activity of neurons, which in turn can lead to alterations in the BBB.^150^, ^154^ As mentioned above, neurons express PD‐1, to which astrocytes and microglia can signal via PD‐L1 expression.^127^, ^128^, ^129^ Neuronal PD‐1 signaling does not induce apoptosis, but instead hyperpolarizes the neuronal membrane to inhibit action potential frequency.^129^ This in turn could lead to a reduction in activity‐dependent transport across the BBB.^155^ Neurons also produce Wnt ligands which reduce BBB permeability by increasing tight junction proteins in ECs.^156^ While neurons partially modulate the BBB via activity, they largely rely on communication with other NVU members (Figure 2). While more information is necessary to determine how neurons might control the BBB, neurons represent an unlikely target for BBB permeability modulation given their moderate impact on the BBB and other essential functions.

## 8 FUTURE DIRECTIONS

Each cellular component of the NVU has both unique and overlapping roles in maintaining BBB permeability. ECs are the initial barrier to the periphery, reinforced with tight junctions, specialized to transport material between the periphery and the CNS. Pericytes, and to some extent, microglia, regulate vascular flow and, while they may also have a structural role, pericytes provide critical trophic support to ECs. Astrocytes, and potentially neurons, are critical to the formation of the BBB, aiding in tight junction formation. Microglia and neurons also potentially modulate BBB permeability through complex signaling networks, although this is an emerging field. The intricate dynamics of BBB formation, function, and maintenance has created a literal and figurative barrier when it comes to therapy development for the treatment of chronic neurologic diseases such as glioblastoma, MS, and AD. While it is possible to increase the permeability of the BBB, this typically results in detrimental off target effects. The BBB is disrupted in many neurological diseases, thus additional interventional disruption would likely prove deleterious as unwanted CNS “intruders” including immune cells and large molecules are able to enter the CNS without proper scrutiny, leading to a cascade of osmotic, trophic, and inflammatory dysregulation. Thus, optimizing targeted and temporary entry of therapeutics into the CNS with minimal BBB dysregulation is the holy grail of next‐generation neurotherapeutics. Promising modalities include encapsulating drugs of interest in a form that allows them to be transported through the BBB and astrocyte endfeet or creating a transient passage that is quickly repaired. Both of these methods are active areas of research using nanoparticles and ultrasonic disruption of the BBB, respectively.^12^, ^157^, ^158^ Ultimately, a deeper understanding of how each of the NVU components modulate CNS drug accessibility may shed new light on actionable therapeutic modalities.

## AUTHOR CONTRIBUTIONS

All authors contributed to the writing, editing, and review of the manuscript.

## CONFLICT OF INTEREST

The authors declare no competing financial interests.

## Data Availability

There are no data in this manuscript.
